# Drug use patterns at a Kenyan referral hospital: A cross-sectional study using WHO core indicators

**DOI:** 10.1371/journal.pone.0354373

**Published:** 2026-07-29

**Authors:** Robi Cynthia Chacha, Ermias Mergia Terefe

**Affiliations:** Department of Pharmacology, Pharmacognosy and Pharmaceutical Chemistry, School of Pharmacy and Health Sciences, United States International University-Africa, Nairobi, Kenya; Faculty of Pharmacy - Istinye University, TÜRKIYE

## Abstract

**Background:**

Suboptimal medicine use practices remain a major global health challenge, particularly in low- and middle-income countries, where it contributes to increased healthcare costs, and the growing burden of antimicrobial resistance. This study aimed to evaluate drug use patterns at Thika Level 5 Hospital in Kenya using World Health Organization (WHO) core drug use indicators.

**Methods:**

A facility-based cross-sectional study was conducted in the outpatient department. Data were collected from 600 prescriptions, 100 patient encounters, and facility-level assessments using standardized WHO/INRUD data collection tools. Descriptive and inferential analyses were performed using IBM SPSS Statistics (version 29), and findings were descriptively compared with WHO reference standards.

**Results:**

The mean number of drugs per prescription was 3.18, which was higher than the WHO recommended range of 1.6–1.8. Generic prescribing was 45.86% while antibiotic prescribing was observed in 58.7% of encounters. Injection use was 13.0% and 76.86% of prescribed medicines were from the Essential Medicines List (EML).

Patient-care indicators showed a mean consultation time of 4.26 minutes and a mean dispensing time of 122.8 seconds, which was below the WHO- recommended reference value. Only 30.0% of medicines were adequately labelled, and 44.0% of patients demonstrated complete knowledge of their medications. Significant associations were observed between dispensing time and patient knowledge (p = 0.001), as well as between labelling adequacy and patient understanding (p < 0.001).

In contrast, all facility indicators met WHO standards, with full availability of essential medicines, treatment guidelines, and stock records.

**Conclusion:**

Despite strong facility readiness, important gaps were identified in prescribing and patient-care practices. These findings highlight opportunities for interventions aimed at strengthening antimicrobial stewardship initiatives, promoting generic prescribing, improving dispensing practices, and enhancing patient counselling to support improved medicine use practices..

## 1 Introduction

The rational use of medicines (RUM) is a cornerstone of effective healthcare delivery, ensuring that patients receive medications appropriate to their clinical needs, in correct doses, for an adequate duration, and at the lowest possible cost to both individuals and health systems [1]. Despite its critical role in improving patient outcomes, suboptimal medicine use practice remains a major global health challenge. This issue is particularly pronounced in low- and middle-income countries (LMICs), where it contributes to increased morbidity, mortality, antimicrobial resistance (AMR), and unnecessary healthcare expenditure. Recent global analyses continue to indicate that a substantial proportion of medicines are prescribed or used inappropriately, highlighting persistent gaps in prescribing and dispensing practices [2,3].

In this study, rational medicine use refers to the appropriate prescribing, dispensing, and use of medicines according to patients’ clinical needs, in correct doses, for adequate duration, and at the lowest possible cost. Polypharmacy was operationally defined as the prescribing of multiple medicines per patient encounter, particularly when the average number of medicines per prescription exceeds the WHO/INRUD recommended range of 1.6–1.8 medicines per encounter.

To support the evaluation of medicine use practices, the World Health Organization (WHO), in collaboration with the International Network for Rational Use of Drugs (INRUD), developed standardized core drug use indicators. These indicators provide objective measures across three domains: prescribing practices, patient-care practices, and facility-level readiness [4]. They are widely used in health systems research to identify patterns of suboptimal medicine use practices and to guide targeted interventions. Evidence from multiple settings continues to affirm their relevance in assessing healthcare quality, particularly in LMICs where health systems often face constraints related to resources, training, and regulatory enforcement [4–8]. **Table 1** presents the WHO/INRUD core drug use indicators and their corresponding optimal values.

**Table 1:** WHO/INRUD core drug use indicators.

Category	Indicator	Optimal Value
**Prescribing Indicators**	Average number of drugs per encounter	1.6–1.8
	% of drugs prescribed by generic name	100%
	% of encounters with an antibiotic prescribed	≤30%
	% of encounters with an injection prescribed	≤10%
	% of drugs prescribed from Essential Medicines List	100%
**Patient-Care Indicators**	Average consultation time	≥3–5 minutes
	Average dispensing time	≥180 seconds
	% of drugs actually dispensed	100%
	% of drugs adequately labeled	100%
	Patients’ knowledge of correct dosage	100%
**Facility Indicators**	Availability of EML and STGs	100%
	Availability of key essential drugs and stock records	100%

Evidence from sub-Saharan Africa consistently demonstrates substantial deviations from WHO-recommended standards. Studies conducted in Kenya, Ethiopia, and other African countries report persistent challenges such as polypharmacy, low rates of generic prescribing, and excessive use of antibiotics and injections [9–11]. These patterns are particularly concerning in the context of the growing burden of antimicrobial resistance, which remains a major public health threat. Recent global estimates indicate that AMR continues to increase, disproportionately affecting LMICs due to inappropriate antibiotic use and limited implementation of antimicrobial stewardship programs [12]. In Kenya, antimicrobial resistance has emerged as a growing public health concern, prompting national efforts to strengthen antimicrobial stewardship and rational medicine use practices.

In addition to prescribing challenges, deficiencies in patient-care practices—such as short consultation and dispensing times, inadequate medication labeling, and poor patient knowledge of drug use—have been widely documented. These factors may negatively influence medication adherence and therapeutic outcomes [13]. Furthermore, facility-related challenges, including inconsistent availability of essential medicines and limited access to standard treatment guidelines, continue to undermine rational medicine use in many healthcare settings [14].

In Kenya, previous studies applying WHO drug use indicators have predominantly focused on prescribing practices, often overlooking patient-care and facility-level dimensions. At Thika Level 5 Hospital, earlier assessments were limited to prescribing indicators, leaving important gaps in understanding the broader context of medicine use. Given that rational medicine use is influenced by interconnected factors spanning prescribing behaviour, patient interaction, and facility readiness, a comprehensive evaluation across all three WHO indicator domains is essential.

Although several Kenyan studies have evaluated prescribing indicators using WHO/INRUD methodology, few have comprehensively integrated prescribing, patient-care, and facility indicators within a single referral hospital setting. Furthermore, limited evidence exists regarding how structural facility readiness relates to actual prescribing and patient-care practices in Kenyan public referral hospitals. This study therefore provides a holistic evaluation of medicine use practices and examines the relationship between facility readiness and clinical implementation of rational medicine use practices.

In Kenyan referral hospitals, medicine use practices may be influenced by high outpatient attendance, referrals from lower-level facilities, limited consultation time, workforce constraints, long patient queues, and pressure to manage complex cases rapidly. Behavioural factors, including habitual prescribing, prescriber preference for brand-name medicines, patient expectations, and incomplete adherence to standard treatment guidelines, may further affect rational medicine use.

We hypothesized that although Thika Level 5 Hospital had adequate facility readiness, gaps would persist in prescribing and patient-care indicators because clinical practice is also influenced by workflow, behavioural, and health-system factors.

Therefore, this study aimed to conduct a comprehensive assessment of drug use patterns at Thika Level 5 Hospital using WHO core drug use indicators. By simultaneously evaluating prescribing, patient-care, and facility indicators, the study seeks to provide a holistic understanding of medicine use practices and generate evidence to inform targeted interventions for improving patient safety, treatment outcomes, and health system efficiency.

## 2 Methods

### 2.1 Study period

The study was conducted between March and April 2026. Data collection was carried out in March 2026, followed by data analysis in April 2026.

### 2.2 Study design and setting

A facility-based cross-sectional descriptive study was conducted to evaluate drug use patterns at Thika Level 5 Hospital, a major public referral hospital in Kenya. The study was carried out in the outpatient department, which manages a high volume of patient consultations and prescription dispensing.

This study design was selected to provide a cross-sectional snapshot of prescribing practices, patient-care interactions, and facility readiness using standardized World Health Organization (WHO) and International Network for Rational Use of Drugs (INRUD) core drug use indicators.

### 2.3 Study population

The study population comprised three components:

1. Outpatient prescriptions issued and dispensed during the study period;
2. Patients attending the outpatient pharmacy for medication dispensing; and
3. Facility-level resources and records relevant to medicine use, including the Essential Medicines List (EML), Standard Treatment Guidelines (STGs), and stock records.

These components were included to enable a comprehensive assessment of prescribing, patient-care, and facility indicators as defined by WHO/INRUD.

### 2.4 Sample size and sampling technique

The sample size was determined based on WHO/INRUD recommendations for drug use indicator studies. A total of 600 outpatient prescriptions were included for the assessment of prescribing indicators. For patient-care indicators, 100 patient encounters were observed and evaluated through direct observation and structured exit interviews. Facility indicators were assessed using a structured checklist of key parameters.

Systematic random sampling was used to select prescriptions from daily outpatient records. After selection of the first eligible prescription, every fifth eligible prescription was reviewed until the required sample size of 600 prescriptions was achieved.For patient-care indicators, every third eligible patient was approached to ensure systematic sampling across outpatient pharmacy flow, reduce selection bias, and allow representation of patients attending at different times during the data collection period.Facility indicators were assessed through direct observation and verification of relevant documents, including medicine availability and access to treatment guidelines.

The selected sample sizes were consistent with WHO/INRUD recommendations for health facility drug use indicator studies and were considered adequate to provide representative estimates of medicine use practices within the outpatient department.

### 2.5 Data collection procedures

Data were collected using standardized WHO/INRUD drug use indicator forms. Prescribing indicators were obtained through retrospective review of selected outpatient prescriptions. Patient-care indicators were collected through direct observation of dispensing practices and structured exit interviews with patients receiving medications at the outpatient pharmacy. The structured exit interview tool was adapted from the WHO/INRUD patient-care indicator form and administered in English or Kiswahili depending on participant preference. The tool assessed whether patients could correctly state the dosage frequency and treatment duration of their dispensed medicines. Responses were compared with the prescription instructions and classified as correct only when the patient accurately stated the relevant information without prompting. The tool was reviewed for clarity before data collection, and data collectors were trained to administer it consistently. Facility indicators were assessed using a structured checklist to verify the availability of essential medicines, treatment guidelines, and stock management records.

Data collection was conducted over the study period by trained data collectors to ensure consistency, accuracy, and reliability. Pharmacy dispensers were aware that an observational medicine use assessment was being conducted; however, data collection was conducted over several days during routine service delivery to minimize behavioural change associated with observation. The data collectors did not interfere with normal pharmacy workflow. Prior to data collection, the data collectors received standardized training on the use of WHO/INRUD data collection tools and study procedures. Standardized procedures were followed throughout the data collection process to minimize variability and enhance data quality. Supervisory review was also conducted during the data collection process to promote consistency in data recording and interpretation.

### 2.6 Study variables and indicators

The study evaluated WHO/INRUD core drug use indicators across three domains: prescribing, patient-care, and facility indicators [15].

Prescribing indicators included the average number of drugs per prescription, percentage of drugs prescribed by generic name, percentage of encounters with an antibiotic prescribed, percentage of encounters with an injection prescribed, and percentage of drugs prescribed from the Essential Medicines List (EML).

Patient-care indicators included average consultation time, average dispensing time, percentage of drugs actually dispensed, percentage of drugs adequately labeled, and patient knowledge of medication use, specifically regarding dosage and duration. Adequate labeling was defined according to WHO/INRUD criteria as inclusion of at least the patient name, medicine name, dosage instructions, frequency, and duration of treatment on the medication label.

Facility indicators included the availability of the Essential Medicines List (EML), availability of Standard Treatment Guidelines (STGs), availability of key tracer drugs, and the presence of stock records.

### 2.7 Data analysis

Data were entered into Microsoft Excel and analysed using IBM SPSS Statistics (version 29). Descriptive statistics—including means, standard deviations, frequencies, and percentages, and 95% confidence intervals where appropriate were used to summarize the data. The observed findings were descriptively compared with WHO reference values to evaluate medicine use practices**.**

Normality of continuous variables was assessed using both the Shapiro–Wilk and Kolmogorov–Smirnov tests. As key variables did not follow a normal distribution, non-parametric statistical methods were applied where appropriate. Inferential analyses included chi-square tests to assess associations between categorical variables and Mann–Whitney U tests to compare differences between groups.

Correlation analyses were performed to evaluate relationships between continuous variables, using Pearson’s correlation coefficient for normally distributed data and Spearman’s rank correlation coefficient for non-normally distributed data. Inferential analyses were limited to assessing relationships within the study dataset and did not involve hypothesis testing against WHO reference benchmarks. All statistical tests were two-tailed, and a p-value of <0.05 was considered statistically significant.

### 2.8 Ethical considerations

Ethical approval for the study was obtained from the United States International University–Africa Institutional Review Board (USIU-A/ISERC/US1297–2026) and the National Commission for Science, Technology and Innovation (NACOSTI/P/26/4185450). Permission to conduct the study was also granted by the management of Thika Level 5 Hospital.

Written informed consent was obtained from all patients who participated in the exit interviews. Participation was voluntary, and respondents were informed of their right to withdraw from the study at any time without any consequences.

Confidentiality and anonymity were strictly maintained throughout the study. No personal identifiers were collected, and all data were handled in a secure and confidential manner.

The study was conducted in accordance with established ethical principles, including respect for persons, beneficence, and justice.

## 3 Results

### 3.1 Study characteristics

A total of 600 outpatient prescriptions were included in the analysis of prescribing indicators, alongside 100 patient encounters evaluated for patient-care indicators. In addition, a facility-level assessment was conducted using a structured checklist to evaluate the availability of essential medicines, treatment guidelines, and stock management practices.

The prescriptions analysed were obtained from the outpatient department and reflect routine clinical encounters during the study period. Data on patient-care indicators were collected through direct observation of dispensing practices and structured exit interviews with patients receiving medications at the outpatient pharmacy.

The prescribing dataset demonstrated variability in the number of drugs per prescription (range: 1–8), indicating heterogeneous prescribing patterns across clinical encounters. Similarly, patient-care indicators showed variability in consultation and dispensing times, reflecting differences in clinical workflow and dispensing practices.

All collected data were complete and included in the final analysis, with no missing values requiring imputation. The sample sizes were consistent with WHO recommendations for drug use indicator studies, supporting the reliability and representativeness of the findings.

### 3.2 Prescribing indicators

#### 3.2.1 Descriptive statistics.

A total of 600 outpatient prescriptions were analysed. The mean number of drugs per prescription was 3.18 (SD = 1.25; median = 3.00; range: 1–8), indicating considerable variability in prescribing patterns. The mean number of drugs prescribed by generic name was 1.46 (SD = 1.23), corresponding to an overall generic prescribing rate of 45.86% (95% CI: 41.9%–49.8%)**.**

Antibiotics were prescribed in 58.7% (352/600) (95% CI: 54.7%–62.6%) of encounters, while injections were prescribed in 13.0% (78/600) (95% CI: 10.5%–15.9%). The mean number of drugs prescribed from the Essential Medicines List (EML) was 2.45 (SD = 1.19), resulting in an overall EML adherence rate of 76.86% (95% CI: 73.3%–80.1%). **Table 2** presents the descriptive statistics for the key prescribing indicators.

**Table 2:** Descriptive statistics of prescribing indicators (n = 600).

Variable	Mean	Median	SD	Range
Number of drugs per prescription	3.18	3.00	1.25	1–8
Number of generic drugs	1.46	1.00	1.23	0–6
Number of EML drugs	2.45	2.00	1.19	0–8

#### 3.2.2 Distribution and normality.

The distribution of the number of drugs per prescription exhibited mild positive skewness (skewness = 0.285). Tests of normality indicated that the data were not normally distributed. The Kolmogorov–Smirnov test yielded a statistically significant result (D = 0.155, p < 0.001), which was corroborated by the Shapiro–Wilk test (W = 0.932, p < 0.001). These findings supported the use of non-parametric statistical methods for subsequent inferential analyses.

#### 3.2.3 Inferential analysis.

Inferential analyses revealed significant relationships among key prescribing variables. A chi-square test of independence demonstrated a statistically significant association between antibiotic prescribing and injection use (χ² (1, N = 600) = 26.19, p < 0.001), with a Cramér’s V of 0.209 indicating a small-to-moderate effect size. Specifically, prescriptions that included injections were less likely to contain antibiotics, which may suggest differences in the clinical contexts in which these medications were prescribed. **Table 3** summarizes the association between antibiotic and injection prescribing.

**Table 3:** Association between antibiotic and injection prescribing (n = 600).

Variable	Antibiotic (%)	No Antibiotic (%)	p-value
Injection prescribed	7.1%	21.4%	<0.001

Correlation analysis demonstrated a strong positive relationship between the total number of drugs per prescription and the number of drugs prescribed from the Essential Medicines List (EML). Pearson’s correlation coefficient was 0.753 (p < 0.001), while Spearman’s rank correlation coefficient was 0.735 (p < 0.001), indicating that prescriptions with a higher number of drugs tended to include more EML-listed medicines.

Further analysis using the Mann–Whitney U test showed that prescriptions containing antibiotics were associated with significantly higher numbers of drugs compared to those without antibiotics (Z = –5.451, p < 0.001). Similarly, antibiotic-containing prescriptions had significantly higher numbers of generic drugs (Z = –5.451, p < 0.001) and EML drugs (Z = –8.703, p < 0.001), which may indicate relatively more complex prescribing patterns in these encounters.

#### 3.2.4 Comparison with WHO standards.

Comparison of the observed prescribing indicators with WHO reference values revealed substantial deviations across multiple parameters. The mean number of drugs per prescription (3.18) was higher than the WHO-recommended range of 1.6–1.8, indicating a pattern of polypharmacy. The proportion of encounters with antibiotics (58.7%) was also above the WHO threshold of ≤30%, while injection use (13.0%) slightly exceeded the recommended maximum of ≤10%

In contrast, the rate of generic prescribing (45.86%) was below the WHO target of 100%, and adherence to the Essential Medicines List (EML) (76.86%) was likewise below the recommended benchmark. These findings collectively indicate substantial departures from recommended standards for medicine use practices. **Table 4** summarizes the comparison between observed prescribing indicators and WHO standards.

**Table 4:** Comparison of prescribing indicators with WHO standards.

Indicator	Observed Value	WHO Standard	Interpretation
Average number of drugs per prescription	3.18	1.6–1.8	Higher than recommended range
Generic prescribing (%)	45.86%	100%	Below recommended target
Antibiotic use (%)	58.7%	≤30%	Above recommended threshold
Injection use (%)	13.0%	≤10%	Slightly above recommended threshold
EML prescribing (%)	76.86%	100%	Below recommended target

### 3.3 Patient-care indicators

#### 3.3.1 Descriptive results.

A total of 100 patient encounters were evaluated for patient-care indicators. The mean consultation time was 4.26 minutes (SD = 1.26; range: 2.1–7.5), while the mean dispensing time was 122.8 seconds (SD = 35.6), indicating variability in the duration of patient–provider interactions.

A high proportion of prescribed medicines (93.0% [95% CI: 86.1%–97.1%]) were actually dispensed to patients. However, only 30.0% (95% CI: 21.2%–40.4%) of dispensed medicines were adequately labelled in accordance with WHO criteria. Assessment of patient knowledge revealed that 69.0% (95% CI: 59.0%–77.7%) of patients correctly identified the dosage frequency of their medications, whereas only 46.0% (95% CI: 36.0%–56.3%) correctly identified the duration of treatment. Overall, 44.0% (95% CI: 34.1%–54.3%) of patients demonstrated complete knowledge of both dosage and treatment duration. Table 5 summarizes the descriptive statistics for patient-care indicators.

**Table 5:** Descriptive statistics of patient-care indicators (n = 100).

Indicator	Mean (SD)/ %	WHO Standard
Consultation time (minutes)	4.26 (1.26)	≥3–5 min
Dispensing time (seconds)	122.8 (35.6)	≥180 sec
Drugs actually dispensed (%)	93.0%	100%
Drugs adequately labelled (%)	30.0%	100%
Patients with correct dosage frequency knowledge (%)	69.0%	100%
Patients with correct treatment duration knowledge (%)	46.0%	100%
Patients with complete knowledge (%)	44.0%	100%

#### 3.3.2 Associations (dispensing time, labelling, and knowledge).

Inferential analysis demonstrated significant relationships between dispensing practices and patient knowledge. Patients who demonstrated complete knowledge of their medications had significantly longer dispensing times compared to those without complete knowledge, as determined by the Mann–Whitney U test (Z ≈ –3.21, p = 0.001). This finding suggests that longer dispensing time may be associated with better patient understanding of medication use.

A chi-square test revealed a statistically significant association between adequate labelling and patient knowledge (χ² (1, N = 100) ≈ 12.45, p < 0.001). Patients who received adequately labelled medications were more likely to correctly identify both the dosage and duration of treatment compared to those who received inadequately labelled medicines. **Table 6** summarizes the association between labelling adequacy and patient knowledge.

**Table 6:** Association between labelling and patient knowledge (n = 100).

Variable	Complete Knowledge (%)	Incomplete Knowledge (%)	p-value
Adequate labelling	Higher proportion	Lower proportion	<0.001

These findings indicate that both dispensing time and labelling quality may play an important role in patient understanding of medication use.

#### 3.3.3 Comparison with WHO standards.

Comparison of patient-care indicators with WHO reference standards revealed substantial deviations across multiple domains. The mean consultation time (4.26 minutes) was within the acceptable range (≥3–5 minutes), although some consultations fell below the minimum recommended threshold. In contrast, the mean dispensing time (122.8 seconds) was below the WHO-recommended minimum of 180 seconds (p < 0.001).

The proportion of prescribed medicines actually dispensed (93.0%) approached the ideal value but remained below the WHO target of 100%. Adequate labelling of dispensed medicines (30.0%) was substantially below the WHO benchmark. Similarly, patient knowledge indicators were below expected standards, with both dosage knowledge (69.0%) and duration knowledge (46.0%) falling below the WHO target of 100%. **Table 7** presents the comparison of patient-care indicators with WHO standards.

**Table 7:** Comparison of patient-care indicators with WHO standards.

Indicator	Observed Value	WHO Standard	Interpretation
Consultation time	4.26 min	≥3–5 min	Within recommended range
Dispensing time	122.8 sec	≥180 sec	Below recommended threshold
Drugs actually dispensed (%)	93.0%	100%	Slightly below recommended target
Drugs adequately labelled (%)	30.0%	100%	Substantially below recommended target
Dosage knowledge (%)	69.0%	100%	Below recommended target
Duration knowledge (%)	46.0%	100%	Below recommended target

### 3.4 Facility indicators

#### 3.4.1 Descriptive findings.

All assessed facility indicators met WHO-recommended standards. The Essential Medicines List (EML) and Standard Treatment Guidelines (STGs) were available and accessible at the point of care. In addition, all selected key tracer drugs were available at the time of assessment, resulting in 100% availability, which was above the WHO minimum threshold of ≥80%. Stock records were also present and appropriately maintained, suggesting effective inventory management practices. **Table 8** summarizes the findings for facility indicators.

**Table 8:** Facility indicator findings.

Indicator	Observed Status	WHO Standard
Essential Medicines List (EML) availability	Available	Available
Standard Treatment Guidelines (STGs) availability	Available	Available
Key tracer drugs availability	100%	≥80%
Stock records	Available	Available

The consistent compliance observed across all facility indicators suggests strong structural capacity to support rational medicine use at the facility level.

### 3.5 Integrated findings across domains

Analysis across the three WHO indicator domains revealed a distinct pattern. While facility indicators demonstrated full compliance with WHO standards, both prescribing and patient-care indicators showed substantial deviations. The coexistence of optimal facility readiness with suboptimal prescribing practices such as polypharmacy, low rates of generic prescribing, and high antibiotic use suggests that the availability of essential resources does not necessarily translate into optimal prescribing practices.

Similarly, deficiencies in patient-care indicators, particularly in dispensing time, medication labelling, and patient knowledge, may reflect gaps in the delivery of pharmaceutical care despite adequate facility infrastructure. The significant associations observed between dispensing time and patient knowledge, as well as between labelling quality and patient understanding, further emphasize the critical role of provider–patient interactions in supporting appropriate medication use.

Collectively, these findings highlight a disconnect between structural capacity and clinical practice, suggesting that behavioural, educational, and workflow-related factors may contribute substantially to medicine use patterns beyond resource availability alone.

### 3.6 Comparison with previous studies

Comparison with previous studies conducted at Thika Level 5 Hospital reveals both consistencies and new insights. Earlier assessments, by Patel and Terefe [16], at the facility primarily focused on prescribing indicators and reported deviations from WHO standards in areas such as polypharmacy, generic prescribing, and antibiotic use. The findings of the present study corroborate these earlier reports, suggesting that suboptimal prescribing practices have persisted over time.

However, unlike previous studies, the current study provides a comprehensive evaluation across all three WHO indicator domains. The inclusion of patient-care and facility indicators demonstrates that prescribing challenges coexist with notable deficiencies in pharmaceutical care practices, particularly in medication labelling and patient knowledge. At the same time, the strong performance observed in facility indicators suggests that the structural prerequisites for rational medicine use are in place.

These findings suggest that while prior interventions may have addressed certain aspects of prescribing behaviour, a more integrated and multidisciplinary approach may be needed to achieve meaningful improvements in overall medicine use. By incorporating patient-care and facility-level perspectives, this study offers a more holistic understanding of drug use patterns at the facility and identifies critical areas that have previously been underexplored.

## 4 Discussion

### 4.1 Overview

This study applied the WHO/INRUD core drug use indicator framework to conduct a comprehensive three-domain evaluation of outpatient medicine use at Thika Level 5 Hospital, Kenya. The findings reveal a consistent and clinically significant pattern: while the facility demonstrated full compliance across all facility indicators, prescribing and patient-care practices deviated substantially from WHO reference standards. This discussion contextualizes each observed finding within the existing literature, explores potential contributing factors and outlines implications for clinical practice, public health policy, and health systems design.

#### 4.1.1 Average number of drugs per prescription.

The mean number of drugs per prescription was 3.18 (SD = 1.25), which was higher than the WHO-recommended range of 1.6–1.8 drugs per encounter. This finding indicates a pattern of polypharmacy and represents the most pronounced deviation from international benchmarks observed in this study. The reported value is higher than those documented in comparable settings; Nyabuti et al. [17] reported a mean of 2.9 drugs per encounter in public primary healthcare centres in Kisii County, Kenya, while Wendie et al. [18] reported a mean of 2.1 in public health centres in Dessie, Ethiopia. Furthermore, a systematic review by Mekonnen et al. [19] across Ethiopian health facilities found that the average number of drugs per encounter ranged from 0.98 to 2.5, suggesting that polypharmacy in sub-Saharan African outpatient settings is a widespread systemic issue rather than a facility-specific anomaly.

Several factors may explain the elevated prescribing rate observed at Thika Level 5 Hospital. As a major referral facility, it manages patients with multiple comorbidities and complex clinical conditions that may necessitate the use of multiple medications across different therapeutic classes. However, the extent of deviation from WHO standards highlights the need for targeted institutional interventions. Polypharmacy is well documented to increase the risk of adverse drug reactions, drug–drug interactions, medication errors, and poor patient adherence, all of which can compromise treatment outcomes.

To address this challenge, the integration of clinical pharmacist-led medication review and de-prescribing initiatives within outpatient care may be beneficial. Such interventions have been shown to optimize prescribing practices by reducing unnecessary medications while maintaining therapeutic effectiveness. Strengthening multidisciplinary collaboration and promoting rational prescribing practices may help mitigate the risks associated with polypharmacy in this setting.

Unlike many previous WHO indicator studies that focused primarily on prescribing patterns, this study suggests that optimal structural readiness does not necessarily translate into rational medicine use practices. This highlights the importance of behavioural and workflow-related determinants in medicine use

#### 4.1.2 Percentage of drugs prescribed by generic name.

The generic prescribing rate observed in this study was 45.86%, which was below the WHO-recommended ideal of 100% and Kenya’s national policy mandate promoting generic-first prescribing in public healthcare facilities. This finding highlights a significant gap in adherence to rational prescribing standards. Comparatively, this rate is higher than the 27.7% reported by Nyabuti et al. [17] in Kisii County, Kenya, but considerably lower than the mean of 91.6% reported in a systematic review across Ethiopian health facilities by Mohammed and Faris [20], suggesting stronger policy implementation in those settings.

Low rates of generic prescribing in public healthcare systems are influenced by multiple factors. Prescribing behaviors are often shaped by habits developed during undergraduate and postgraduate training, as well as by patient preferences for branded medications. Additionally, misconceptions regarding the quality and efficacy of generic medicines, coupled with limited awareness of bioequivalence standards among clinicians, may further contribute to suboptimal uptake of generic prescribing practices.

From a health systems perspective, reliance on brand-name prescribing has significant economic implications. It increases the financial burden on both patients and healthcare systems, potentially limiting access to essential medicines and undermining supply chain efficiency. Addressing this challenge requires targeted institutional interventions. The Drugs and Therapeutics Committee, in alignment with Ministry of Health regulatory frameworks, could consider implementing structured prescriber education programs, reinforce adherence to standard treatment guidelines, and strengthen formulary enforcement mechanisms. Such measures may help support more appropriate prescribing practices and improve adherence to generic prescribing recommendations.

#### 4.1.3 Percentage of encounters with an antibiotic prescribed.

Antibiotics were prescribed in 58.7% of outpatient encounters, nearly double the WHO-recommended threshold of ≤30%. This represents one of the largest deviations among the prescribing indicators assessed in this study. Although this rate is lower than the 84.8% reported by Nyabuti et al. [17] in Kisii County, Kenya, and broadly comparable to the 43.9% documented by Wendie et al. [18] in Dessie, Ethiopia, it remains substantially above evidence-based benchmarks. These findings reinforce the persistence of high rates of antibiotic prescribing as a systemic challenge across healthcare settings in the region.

The public health implications of this pattern are considerable. Murray et al. [21] estimated that antimicrobial resistance (AMR) was associated with approximately 1.27 million deaths globally in 2019, with sub-Saharan Africa bearing a disproportionate share of this burden. In response, Kenya’s National Action Plan on AMR identifies inappropriate outpatient antibiotic prescribing as a potential contributor to resistance and emphasizes the need for strengthened antimicrobial stewardship across all levels of care.

The statistically significant negative association observed between antibiotic and injection prescribing (χ² = 26.191, p < 0.001; Cramér’s V = 0.209) suggests that these prescribing practices may occur within distinct clinical contexts. Specifically, prescriptions involving injections were less likely to include antibiotics, potentially reflecting clinician differentiation between infectious and non-infectious conditions. However, further qualitative investigation is needed to better understand the underlying decision-making processes.

Addressing excessive antibiotic use may require strengthened implementation of structured antimicrobial stewardship interventions. These should include guideline-based prescribing, pre-authorization protocols for selected broad-spectrum antibiotics, and regular audit-and-feedback mechanisms. Strengthening diagnostic support and promoting continuous professional development for prescribers will also be critical in supporting more appropriate antibiotic prescribing practices and mitigating the growing threat of antimicrobial resistance.

#### 4.1.4 Percentage of encounters with an injection prescribed.

Injections were prescribed in 13.0% of outpatient encounters, slightly exceeding the WHO-recommended threshold of ≤10% [15]. Although this deviation is less pronounced than that observed for antibiotic prescribing, frequent use of injectable formulations may carry important clinical and economic considerations. These may include risk of blood borne infection transmission, injection site complications, and higher direct costs to patients compared to oral alternatives.

Cross-tabulation analysis revealed that prescriptions without antibiotics were more likely to include an injection (21.4%) than those containing antibiotics (7.1%). This pattern may reflect differences in prescribing approaches across varying clinical contexts, particularly where parenteral administration is perceived to provide more rapid therapeutic effects.

The WHO discourages routine use of injectable medications in outpatient settings unless clinically indicated [15]. Therefore, reinforcing adherence to standard treatment guidelines through continuous professional development and targeted prescribing audits may help optimize injection prescribing practices. Promoting appropriate selection of dosage forms based on clinical need may also help reduce unnecessary injection use.

#### 4.1.5 Percentage of drugs prescribed from the essential medicines list.

Adherence to the Essential Medicines List (EML) was 76.86%, representing the closest alignment to WHO standards among the prescribing indicators, yet still falling short of the ideal benchmark of 100%. This finding indicates that approximately one in four prescribed medicines falls outside the national EML, suggesting room for improvement in formulary adherence.

A strong positive correlation was observed between the total number of drugs per prescription and the number of EML drugs prescribed (Pearson r = 0.753; Spearman ρ = 0.735; both p < 0.001), indicating that clinicians generally maintain familiarity with EML recommendations, even in prescriptions involving multiple medications. This finding suggests that deviations from the EML may not solely reflect lack of awareness of formulary recommendations.

Comparatively, Wendie et al. [18] reported 100% adherence to the EML in Dessie, Ethiopia, while Mekonnen et al. [19] found approximately 80% essential medicine availability across Ethiopian facilities. The adherence rate observed in this study is therefore at the lower end of the regional range. Strengthening formulary management through active oversight by the Drugs and Therapeutics Committee, alongside regular updates of the EML based on current evidence and clinical needs, may help improve adherence. Additionally, integrating EML-based prescribing into routine clinical workflows and monitoring systems may further support appropriate medicine use practices.

### 4.2 Patient-care indicators

#### 4.2.1 Average consultation time.

The mean consultation time was 4.26 minutes (SD = 1.26), with a range of 2.1 to 7.5 minutes. Although the WHO does not define a strict minimum consultation duration, existing literature suggests that consultations shorter than 3–5 minutes may be insufficient for comprehensive history-taking, clinical assessment, and appropriate prescribing in outpatient settings [22]. The mean consultation time observed in this study falls within this acceptable range; however, the lower bound of 2.1 minutes recorded in some encounters raises concerns regarding the adequacy of patient evaluation in certain cases. Consultations as short as 2.1 minutes may reflect the realities of a busy Level 5 referral hospital serving a large catchment population. High outpatient attendance, referrals from peripheral facilities, staff workload, triage bottlenecks, and pressure to reduce waiting times may limit opportunities for comprehensive assessment, medication review, and patient counselling

These findings are consistent with broader evidence from sub-Saharan Africa. A multi-country analysis reported average consultation times ranging from 2 to 6 minutes in public sector outpatient departments substantially shorter than those typically observed in high-income settings [23]. Such constraints may be influenced by systemic factors, including high patient volumes, limited healthcare workforce capacity, and inefficient appointment or triage systems.

Therefore, efforts to improve consultation quality at Thika Level 5 Hospital should focus on addressing these structural challenges rather than attributing deficiencies solely to individual clinician performance. Interventions such as workflow optimization, task-shifting, and improved patient flow management may help support adequate consultation time without compromising service delivery efficiency.

#### 4.2.2 Average dispensing time.

The mean dispensing time was 122.8 seconds (SD = 35.6), which was below the WHO-recommended minimum of approximately 180 seconds. This duration is considered necessary for dispensers to adequately review prescriptions, provide medication instructions, and counsel patients effectively [15].

This finding is consistent with reports from comparable settings. Nyabuti et al. [17] documented a mean dispensing time of 131.5 seconds in Kisii County, Kenya, while Wendie et al. [18] reported 105 seconds in Ethiopia. Similarly, Gidebo et al. [24] found dispensing times ranging from 96 to 152 seconds across hospitals in Southern Ethiopia. The consistency of these findings across different contexts suggests that limited dispensing time may represent a broader challenge in outpatient pharmacy services in low- and middle-income countries rather than an isolated institutional issue.

Importantly, the implications of reduced dispensing time extend beyond operational efficiency. This study demonstrated a statistically significant association between dispensing time and patient knowledge, with patients who had complete understanding of their medications experiencing significantly longer dispensing interactions (Mann–Whitney U test; Z ≈ −3.21, p = 0.001). This finding suggests that longer dispensing interactions may support improved patient understanding of medication use.

In the context of a Level 5 referral hospital, where patients often receive multiple medications for complex conditions, insufficient dispensing time may affect patient understanding of medication instructions and subsequent medication use practices. Addressing this gap may require targeted interventions, including workflow restructuring, increased staffing, and implementation of standardized counselling protocols to help ensure that patients receive adequate medication-related information.

#### 4.2.3 Percentage of drugs adequately labelled.

Only 30.0% of dispensed medicines were adequately labelled according to WHO criteria, which require inclusion of the patient’s name, drug name, dose, frequency, duration, and storage instructions. Although this proportion is slightly higher than the 22.6% reported in Kisii County healthcare centres, it remains markedly below the recommended standard and suggests important gaps in medication labelling practices.

This study identified a statistically significant association between adequate labelling and patient knowledge of medication use (χ² ≈ 12.45, p < 0.001), indicating that labelling quality may play an important role in patient’s ability to correctly manage their medications. Inadequate labelling may increase the likelihood of dosing errors, improper storage, accidental ingestion by household members, and poor medication use practices, particularly among patients with limited health literacy.

In Kenya, the Pharmacy and Poisons Board mandates adequate labelling of all dispensed medicines as a core patient safety standard. The approximately 70% non-compliance observed in this study therefore highlights an important area for institutional quality improvement. Evidence-based interventions such as the use of pre-printed labelling templates, digital label printers, and standardized dispensing and counselling checklists have been shown to improve labelling practices without imposing substantial additional time or resource burdens. Implementing such measures may enhance medication safety and patient understanding in this setting. At Thika Level 5 Hospital, improved labelling could be integrated into the existing pharmacy counter workflow by using preformatted label templates, pre-printed auxiliary labels, and simple label printers positioned near dispensing points. Labels could be prepared while medicines are being retrieved, followed by brief counselling at medicine handover, thereby improving labelling quality without substantially increasing waiting time.

#### 4.2.4 Patient knowledge of medication use.

Patient knowledge of prescribed medications was assessed across two key domains: dosage frequency and treatment duration. The findings indicate that 69.0% of patients correctly identified their dosage frequency, while only 46.0% were able to accurately state the duration of treatment. Overall, only 44.0% of patients demonstrated complete knowledge of both parameters, highlighting a substantial gap in patient understanding that may affect medication use practices.

The observed dosage knowledge rate is higher than the 54.7% reported in Kisii County and findings from Dessie, Ethiopia, which may reflect relatively higher health literacy levels among the urban population served by Thika Level 5 Hospital. However, the markedly low level of knowledge regarding treatment duration is particularly important in the context of antibiotic use.

Incomplete adherence to prescribed antibiotic courses has been associated with the development and spread of antimicrobial resistance. The coexistence of high antibiotic prescribing rates (58.7%) and limited patient understanding of treatment duration observed in this study may therefore represent an important public health concern. Addressing this issue may require targeted and integrated interventions.

Structured patient counselling programs, particularly for antibiotic therapies, may help improve patient understanding of medication instructions. Additionally, the provision of clear, written medication instructions such as take-home information leaflets in both Kiswahili and English may reinforce verbal counselling and support patient comprehension. Strengthening communication between healthcare providers and patients may further support appropriate medication use practices.

### 4.3 Facility indicators

Thika Level 5 Hospital demonstrated full compliance across all four WHO facility indicators assessed. The Essential Medicines List (EML) and Standard Treatment Guidelines (STGs) were available and accessible at the point of care; all selected key tracer drugs were in stock, achieving 100% availability and exceeding the WHO minimum threshold of ≥80%; and stock records were properly maintained. This level of performance compares favourably with findings from similar settings in sub-Saharan Africa. For example, Nyabuti et al. [17] reported that only 20% of facilities in Kisii County, Kenya, had access to a current copy of the Kenya Essential Medicines List, while Wendie et al. [18] documented 64.1% essential medicine availability in Ethiopian health centres. Similarly, Gidebo et al. [24] found that treatment guidelines were available in only approximately 25% of sampled hospitals in Ethiopia.

The observed 100% availability of tracer drugs at Thika Level 5 Hospital is particularly significant from a health systems perspective. Drug stock-outs are known to contribute to off-formulary prescribing, therapeutic substitutions, and reduced patient satisfaction, all of which can negatively affect prescribing and patient-care practices [25]. The consistent availability of essential medicines in this setting may partly explain the relatively high EML adherence rate (76.86%) observed in this study, as prescribers may be more likely to follow formulary recommendations when medicines are reliably accessible. The finding of 100% tracer medicine availability but 76.86% EML prescribing does not represent a contradiction because the two indicators measure different aspects of medicine use. Tracer medicine availability reflects whether selected key medicines were physically available at the facility, whereas EML prescribing assesses whether all medicines prescribed during patient encounters were listed in the Essential Medicines List.

However, it is important to distinguish between structural capacity and behavioural implementation. Despite full compliance with facility indicators, substantial gaps were identified in prescribing and patient-care practices. This disconnect suggests that the mere availability of EML and STG resources alone may not ensure their consistent application in clinical decision-making.

To bridge this gap, targeted interventions may be needed to support translation of structural readiness into improved clinical practice. These may include integrating STG-based prescribing into routine clinical workflows, linking adherence to formulary guidelines with performance evaluation systems, and incorporating drug use indicator monitoring into institutional quality assurance frameworks. Such strategies may help ensure that the strong infrastructural foundation observed at this facility supports appropriate medicine use practices.

### 4.4 Integrated findings across domains

The integration of findings across prescribing, patient-care, and facility domains reveals an important systems-level observation at Thika Level 5 Hospital demonstrates strong structural capacity but suboptimal prescribing and patient-care practices. While facility indicators showed full compliance with WHO standards, substantial deficiencies were identified in prescribing patterns particularly polypharmacy, low rates of generic prescribing, and excessive antibiotic use as well as in patient-care practices, including limited dispensing time, inadequate labelling, and reduced patient knowledge.

This pattern may reflect a broader health systems phenomenon in which improvements in infrastructure do not necessarily translate into enhanced service delivery practices. The significant associations identified in this study particularly between dispensing time and patient knowledge, and between labelling quality and patient understanding highlight the potential importance of provider–patient interactions in supporting appropriate medication use practices. These findings suggest that medicine use practices may be influenced not only by the availability of resources but also by the clinical decision-making processes and the effectiveness of communication during care delivery.

The coexistence of high antibiotic prescribing rates and low patient knowledge of treatment duration is particularly important from a public health perspective. Limited patient understanding of treatment duration may contribute to incomplete adherence to prescribed regimens, which has been associated with antimicrobial resistance. These findings therefore suggest the importance of integrated interventions that address both prescribing practices and patient engagement.

Overall, these findings emphasize the value of adopting a systems-based approach to improving medicine use practices. Effective interventions may need to target multiple levels of care including prescribers, dispensers, and patients while leveraging the existing strong facility infrastructure to support sustainable improvements. Evidence from previous studies suggests that coordinated, multi-component strategies may be more effective than isolated interventions in supporting improved medicine use practices in low- and middle-income country settings.

### 4.5 Public health and clinical implications

The findings of this study have important implications for both clinical practice and public health policy. The high prevalence of polypharmacy and high rate of antibiotic prescribing observed at Thika Level 5 Hospital underscores the need for targeted interventions to support more appropriate prescribing practices. In particular, the elevated rate of antibiotic use may represent an important concern in the context of antimicrobial resistance (AMR), a major global health challenge that disproportionately affects low- and middle-income countries. Strengthening antimicrobial stewardship programs through guideline-based prescribing, routine audit and feedback, and enhanced diagnostic support may help support more appropriate antibiotic use practices.

The low rate of generic prescribing also has important economic implications. Increased reliance on brand-name medicines may increase healthcare costs for both patients and health systems, potentially limiting access to essential treatments. Promoting the use of generic medicines through policy enforcement, prescriber education, and adherence to institutional prescribing guidelines may help improve affordability and support sustainable access to care.

From a clinical perspective, deficiencies in patient-care indicators particularly short dispensing times, inadequate labelling, and limited patient knowledge highlight opportunities to strengthen pharmaceutical care services. The significant associations observed between dispensing time, labelling quality, and patient understanding suggest that potential importance of effective provider–patient communication in supporting appropriate medication use practices. Evidence-based interventions, including standardized counselling protocols, improved labelling practices, and the provision of patient education materials, may help improve patient understanding and reduce the risk of likelihood of medication –related errors.

Importantly, the strong performance in facility indicators provides an opportunity to leverage existing infrastructure to support these improvements. Integrating medicine use improvement initiatives into routine hospital operations such as continuous professional development programs, quality assurance frameworks, and electronic prescribing systems may help support sustainable improvements in medicine use practices and overall health system efficiency.

### 4.6 Strengths and limitations

This study has several notable strengths. First, it provides a comprehensive evaluation of drug use patterns across all three WHO/INRUD core indicator domains prescribing, patient-care, and facility indicators allowing for a holistic assessment of rational medicine use. Second, the relatively large sample size of 600 prescriptions enhances the reliability and robustness of the findings. Third, the inclusion of inferential statistical analyses strengthens the study by identifying significant relationships between variables, particularly within patient-care practices, thereby offering deeper insights beyond descriptive measures.

This study has some limitations. First, its cross-sectional design provides a snapshot of medicine use practices and cannot establish causal relationships. Second, the study was conducted in a single Level 5 referral hospital, which may limit generalizability to other settings. Third, patient knowledge was assessed through exit interviews and may have been affected by recall or social desirability bias. Fourth, the study did not assess the clinical appropriateness of individual prescriptions, particularly antibiotic prescriptions. Finally, seasonal variations in disease patterns and medicine use were not assessed.

## 5 Conclusion

This study provides a comprehensive assessment of drug use patterns at Thika Level 5 Hospital using WHO core drug use indicators, revealing significant gaps in prescribing and patient-care practices despite optimal facility readiness. High levels of polypharmacy, low rates of generic prescribing, and excessive antibiotic use highlight persistent challenges in achieving rational prescribing, while deficiencies in dispensing time, medication labelling, and patient knowledge point to important weaknesses in pharmaceutical care delivery.

The coexistence of strong facility indicators with suboptimal prescribing and patient-care practices suggests the need for interventions that address behavioural and system-level factors in addition to structural capacity. Strengthening antimicrobial stewardship initiatives, promoting generic prescribing, improving dispensing practices, and enhancing patient education may help support improved medicine use practices.

Furthermore, integrating routine drug use monitoring into hospital quality improvement systems may help support sustained improvements in medicine use practices. Bridging the gap between resource availability and clinical practice may contribute to improved healthcare efficiency and support broader public health efforts, including antimicrobial stewardship.
